# Factors associated with neonatal mortality in the general population: evidence from the 2007 Zambia Demographic and Health Survey (ZDHS); a cross sectional study

**DOI:** 10.11604/pamj.2015.20.64.5616

**Published:** 2015-01-23

**Authors:** Etambuyu Lukonga, Charles Michelo

**Affiliations:** 1 Department of Public Health, School of Medicine, University of Zambia, Lusaka, Zambia; 2 Central Statistical Office, Lusaka, Zambia

**Keywords:** Logistic regression, neonatal mortality, determinants

## Abstract

**Introduction:**

Neonatal mortality accounts for almost 40 percent of under-five child mortality globally and this could be associated with a complex chain of factors including but not limited to socio-economic, biological and healthcare-related factors. We examined factors that may be associated with neonatal mortality in Zambia.

**Methods:**

Using across-sectional design, data were extracted from the 2007 Zambia Demographic and Health Survey for women using a “Women's Questionnaire” for respondents aged 15-49 years in the selected households. Records of women who reported having given birth to live infants within the five years preceding the survey defined the study population. However only records on those infants who could have lived through the first month (28 days) were assessed (de facto population).

**Results:**

Overall (n=6 435), there were 3204(49.8%) males and 3231(50.2%)females. There were 219 (3.4%) neonatal deaths recorded. Low birth weight and overweight were reported as the prominent factors. The odds of dying were significantly higher for infants with low birth weight compared to infants born with normal weight, (aOR=2.58, 95%CI 1.02-6.49). The pattern was the same in both rural though insignificant. Over weight born babies showed increased odds of dying (aOR 3.21, 95%CI 1.36-7.59). Compared to infants born from Mothers with no education, infants born from mothers with higher education were associated with increased odds of dying (aOR 3.55, CI 95%, 1.26-9.94).

**Conclusion:**

Neonatal survival is still a challenge in this population and determinants show varying socio-demographic contrasts. This may suggest limitations in past efforts to improve neonatal health. Future strategies need to continue but should account for varying setting specific epidemiological contrasts.

## Introduction

Globally, it is estimated that of the 130 million infants born each year worldwide, 4 million die in the first 28 days of life [1]. The first four weeks of life, the neonatal period, is the most vulnerable time for a child. Despite accounting for almost 40 percent of all under-five child deaths and more than half of infant deaths, neonatal mortality has not been given the priority it deserves [2]. Mortality in the neonatal period tends to decline more slowly than the post-neonatal period (1-59 months), every region of the world is experiencing an increase in the proportion of newborn deaths. As a result, neonatal deaths as a proportion of under-five deaths increased globally from 37 percent in 1990 to 44 percent in 2013 [3]. In 2013, almost 1 million newborns died on the day they were born accounting for 16 percent of all under five deaths and more than a third of all neonatal deaths. A total of 2 million newborns died within the first seven days after birth representing 73 percent of all neonatal deaths [4]. According to the Convention on the Rights of the Child, new- borns have the basic right to enjoy the highest attainable standard of health [5]. The fourth Millenium Development Goal (MDG) entails the reduction of child mortality by two-thirds by the year 2015, from the base year of 1990. However, if the MDG target of a two thirds reduction in child mortality by 2015 is to be achieved [2] neonatal mortality must be addressed.

Studies have indicated that neonatal deaths stem from poor maternal health, inadequate care during pregnancy, inappropriate management of complications during pregnancy and delivery, poor hygiene during delivery and the first critical hours after birth and lack of new born care [6]. Causes of death in the neonatal period in the developing world are poorly measured also, though major components are believed to be birth asphyxia, severe infections, complications of prematurity and tetanus [7]. Neonatal morbidity is still high in developing countries and is due primarily to negligence of female health, nutrition, deliveries by un-skilled personnel and poor antenatal care and this high morbidity significantly contributes to the deaths largely due to poor health care system responses coupled by poor social structures [8]. In order to address neonatal mortality, a clear understanding of the associated risk factors is thus critically necessary.

Neonatal mortality is still a problem in Zambia, estimated to be 34 per 1000 live births according to the 2007 Zambia Demographic and Health Survey (ZDHS) [9]. In another study conducted in Zambia, there were 32 neonatal deaths observed, and 84 percent of these occurred within the first week of life, primarily because of infections and prematurity [10]. The fourth Millennium Development Goal (MDG) seeks the reduction of child mortality by two-thirds by the year 2015. However, if the target is to be achieved in Zambia, the problem has to be investigated so that associated factors and core drivers are understood and inform neonatal survival strategies. A good understanding of these factors is cardinal in guiding the development of focused and health-based high impact interventions that has the potential to significantly reduce the number of neonatal deaths. However, a systematic search on available information has continued to reveal paucity of data on factors influencing neonatal mortality at both community and health facility levels in Zambia. In this study we aimed to investigate and identify population based factors that may be associated with neonatal mortality.

## Methods

***Study Design:*** Data stem from a cross-sectional study that re-analyzed data from the 2007 ZDHS, a nationally representative survey of 7 146 women aged 15-49 years. The ZDHS used standardized methods that have achieved high individual and household response rates. The sample frame was the geographical distribution of household clusters from the 2000 Census of Population and Housing. At least 85 households were in a cluster. With a minimum cluster take of 25 completed interviews of women, 320 clusters were allocated proportional to the population size of provinces within urban and rural areas. One hundred urban and 220 rural clusters were selected at the first stage. At the second stage, households were selected after field listing to update the household information in the selected clusters. De-facto household members of women 15-49 years in 8200 selected households. Details of the ZDHS methodology is reported elsewhere [9]. Using data from the 2007 ZDHS focused on women and utilized “Women′s Questionnaire”, records of women who reported having given birth to live infants within the five years preceding the survey were included as the study population. However, only records of women whose records of their babies the first month (28 days) were available and complete were assessed. All incomplete or missing records were noted but excluded from the final analysis.

***Data Extraction:*** The information recorded and extracted from the Women′s Questionnaire records included the women′s demographic characteristics, their full birth history, history of antenatal care for the most recent birth within a five-year period preceding the survey, delivery and postnatal care for all births, as well as the survival of their live-born infants. In addition, the data obtained from the full birth histories collected from eligible women aged 15-49 years in sampled households during the ZDHS was all extracted. For each record of live birth, the month and year of the birth were extracted and recorded. Given that when and if the child had died, the mother was asked for the age of the child at death in days, months and years during the ZDHS, these deaths reported we extracted and defined neonatal deaths. Proximate determinants at the individual level were extracted by examining and recording available socioeconomic information that could possibly have had an impact on neonatal mortality. These variables included information on maternal age at child birth to represent maternal factors; maternal subjective assessment of the infant's size and infant′s birth rank which represented the neonatal factors; delivery assistance and place of delivery for delivery factors; and antenatal care visits and place of delivery to represent pre-delivery factors.

***Statistical Analysis:*** Data was analysed using STATA version 12 (College Station, Texas, USA) special edition to produce descriptive and inferential statistics. Multiple logistic regression incorporating survey weights were performed to identify the factors associated with neonatal mortality. A p-value of

***Ethics:*** The ZDHS obtained ethical approval from the Tropical Disease and Research Centre (TDRC) in Ndola, Zambia and the US Centre for Disease Control and Prevention (CDC) Atlanta's Research Ethics Review board. Participation in the survey was based on informed and voluntary consent. Participants were informed about this in accordance with ethical requirements. Our re-analysis of the data did not infringe on participants' privacy and was judged by ourselves to pause minimal to no risk since these data were already anonymized, approved and made available for public use and could be used for academic purposes. In addition to the above ethical measures, we sought a waiver from the University of Zambia Biomedical Research Ethics Committee (**ethical approval certificate number 010-03-13**) that granted us permission to conduct the study on factors influencing neonatal mortality based on the 2007 ZDHS.

## Results

### Participation and distribution

Overall (n=6 435), there were 3204(49.8 percent) live born males and 3231(50.2 percent) live born females (Table 1). Over 75% of the baby's mother had no formal education. The response rate for the 2007 ZDHS household questionnaire was 97.8 percent for both rural and urban areas and for eligible women the response rate was 96.5 percent. The main reasons for non-response was absence during the survey period and none refused.

**Table 1:** Overall population and neonatal mortalitydistribution by background characteristics from the women's 2007 Zambia Health and Demographic survey

Predictor Variable	Total Number of Births	Neonatal Death	
Age of Mother		Number	Percent
12-17	2 649	101	46.1
18-24	3 520	103	47.2
25-29	233	14	6.4
30-34	30	0	0
35-49	3	1	0.3
**Birth Order**			
1-3^rd^ birth order	3 502	116	53
4-6^th^ birth order	1 998	76	35
7^th^ + birth order	935	27	12
**Place of Delivery**			
Home	3325	98	45
Government health facility	2745	103	46.9
Private health facility	324	13	5.9
Other places	23	0	0
Unspecified	18	4	2.2
**Assisted to Deliver**			
Skilled doctor/midwife	2 969	111	52.5
Traditional birth attendant	1 441	42	20.1
Relative	1 672	52	24.6
No one	303	5	2.8
**Birth Weight**			
1000-2499	285	17	22
2500-3999	2 384	50	61
4000 +	407	14	17
**ANC Visits**			
1 Antenatal care visit	95	4	4.8
2 Antenatal care visits	336	12	12.7
3 Antenatal care visits	1 068	21	21.6
4+ Antenatal care visits	2 494	61	60.8
**Place of Antenatal Care**			
**Home**	22	1	0.8
Government facility	3 729	92	91.5
Private/Mission	268	7	7.7
Other	20	0	0
**Highest Level of Mother's Education**			
No education	4 089	147	76.7
Primary education	1 320	34	18.2
Higher education	156	9	5.1
**Total**	6 435	219	3.4

### Neonatal mortality

Of all the live-born infants, there were 219 (3.4 percent) neonatal deaths recorded. Below is the percentage distribution of neonatal mortality by background characteristics(Table 1). Table 2 shows adjusted odds ratio of the possible factors associated with neonatal mortality, a result from a multivariate logistic regression. The outcome of neonatal mortality showed statistically significant associations with birth weight, Mother's age at birth and Level of education. The were variations in the odds of neonatal mortality by type of residence (rural/urban). The odds of neonates dying in rural areas were higher than that of urban areas. Babies with low birth weight showed increased odds of dying (AOR 2.58, 95% CI 1.02-6.49) than normal sized babies. The odds of dying for large sized born babies were more than 3 times the odds for normal sized babies. Age of Mother also showed statistically significant associations with neonatal mortality. The odds of dying for babies born from mothers in the age group 18-24 years (AOR 0.47, 95% CI 0.23-0.96) were significantly lower than the odds for babies born from mothers in the age group 12-17 years. The results also showed that there was a correlation between mother's education and neonatal death. Babies born from mothers whose level of education was secondary and higher had increased odds of dying compared to babies born from mothers with no education.

**Table 2:** Factors associated with neonatal mortality in the 2007 Zambia Demographic and Health Survey: Results of multivariate logistic regression

Background Factor		Overall Total		Urban		Rural	
		Prevalence n(%)	aOR 95%CI	Prevalence, n(%)	aOR 95%CI	Prevalence, n(%)	aOR 95%CI
**Mother's Age Group (years)**	12-17	101 (46.1)	1	27 (38.7)	1	74 (49.6)	1
	18-24	103 (47.0)	0.47 (0.23-0.96)	37 (52.3)	0.48 (0.20-1.13)	67 (44.7)	0.52 (0.16-1.66)
	25-29	14 (6.4)	0.83 (0.29-2.35)	6 (9.0)	0.93 (0.30-2.85)	7 (5.1)	0.59 (0.11-2.97)
**Birth Order**	1-3^rd^	116 (53.0)	1	46 (65.6)	1	70 (47.1)	1
	4-6^th^	76 (34.7)	0.91 (0.41-1.99)	19 (26.5)	0.73 (0.25-2.10)	57 (38.6)	1.08 (0.34-3.39)
	7^th^ +	27 (12.3)	1.11 (0.37-3.31)	6 (7.9)	0.92 (0.13-6.07)	21 (14.3)	1.33 (0.33-5.48)
**ANC visits**	1	5 (4.8)	1	1 (1.7)	1	4 (7.0)	1
	2	13 (12.7)	1.03 (0.08-12.3)	6 (13.9)	1.42 (0.11-18.81)	7 (11.9)	-
	3	22 (21.6)	1.06 (0.13-8.46)	6 (14.5)	0.75 (0.79-7.09)	15 (26.6)	2.24 (0.82-6.11
	4	61 (61.0)	0.88 (0.12-6.36)	29 (69.9)	0.93 (0.12-6.91)	33 (54.5)	-
**Place of ANC ( Proxy for quality of Care)**	Home	1 (0.8)	1	0 (0)	1	1 (1.4)	1
	Government health facility	92 (91.5)	0.72 (0.37-1.39)	36 (87.3)	-	56 (94.4)	-
	Private/mission hospital	8 (7.7)	-	5 (12.7)	1.63 (0.78-3.41)	3 (4.2)	1.63 (0.78-3.41)
**Place of delivery**	Home	99 (45.0)	1	17 (23.6)	1	82 (55.0)	1
	Government health facility	103 (47.0)	3.28 (0.45-23.4)	48 (68.9)	0.50 (0.18-1.34)	54 (36.5)	2.26 (0.31-16.22
**Assisted delivery provider**	Skilled health professional	112 (52.5)	1	52 (75.4)	1	59 (41.4)	1
	Relative/Friend	52 (24.6)	1.57 (0.21-11.6)	13 (18.8)	2.87 (0.27-30.62)	39 (27.5)	1.43 (0.22-9.18
	No one	6 (2.8)	2.52 (0.48-13.2)	1 (1.0)	-	5 (3.6)	2.39 (0.33-17.11
**Birth Weight**	Normal BirthWeight	50 (61.0)	1	28 (65.0)	1	21 (56.3)	1
	Low Birth Weight	17 (21.7)	2.58 (1.02-6.49)	11 (24.0)	2.33 (0.71-7.59)	7 (19.0)	3.05 (0.78-11.9)
	Overweight	14 (17.3)	3.21 (1.36-7.59)	5 (11.0)	2.99 (0.87-10.22)	9 (24.7)	3.53 (1.08-11.5)
**Mother's Educational Level**	No Education	147 (76.7)	1	35 (54.5)	1	111 (88.1)	1
	Primary Education	35 (18.2)	1.39 (0.64-3.03)	20 (31.3)	1.12 (0.36-3.44)	15 (11.4)	1.48 (0.43-5.10)
	Higher Education	9 (5.1)	3.55 (1.26-9.94)	9 (14.2)	1.89 (0.48-7.35)	1 (0.5)	8.43 (1.17-60.55)

## Discussion

High neonatal mortality was observed in the records of data from the general population. This mortality was largely associated with low birth weight, place of deliveries as well as higher educational levels for the mothers in addition to presence of insufficient antenatal care attendance as core determinants of neonatal mortality in this population. These determinants were most prominent in rural populations irrespective of the age groups of the mothers. Furthermore, the concentration of burden of neonatal mortality found to be heavier in rural groups may be a further signal of poor socio-economic status in this part of the world. An analysis done by UNICEF shows that lower household wealth, an uneducated mother and birth in rural areas lower a newborn's chances of survival within the first 28 days of life [11]. Socioeconomically, mother's education (secondary or higher level of schooling) had a protective effect against neonatal mortality and this finding is supported worldwide [12–15]. A common supporting argument is that maternal education increases mother's knowledge about child health and health care services [16] and thereby improves health care seeking behaviours for their children and themselves [17]. Contrary to the widely held expectation, however, in this study, increased neonatal mortality risk was associated with higher education levels for mothers. The influence of higher education levels for mothers was associated with higher odds of neonatal deaths than mothers with low education levels. This finding suggests the presence of unmeasured factors. There are possible arbitrating factors not measured such as environmental sanitation/ hygiene; maternal nutrition and the number of mothers with high education levels was small compared to those with low education (Table 1). It is possible that differential mortality patterns among non-participants could have biased our analysis of the exact burden and associated determinants. However there may be no way of estimating the magnitude and direction of this effect in the absence of proxies for non-participants. Firstly we observe that only data relating to women who were alive and age 15 or above were captured. In contrast, those women less than 15 years old or had died were not captured. Assuming that these are risky populations, we could thus conclude that we may have underestimated neonatal mortality burden. Further, we are also aware that in other settings, population based data may not be the best for estimating mortality and as such other methods perceived to be more reliable and giving valid data have been devised. However, we think that the determinants may not be differentially misclassified between participants and non-participants in that they reflect both individual and community factors that may be cross-cutting than individualized. They thus may reflect a community state present not just at a time but most probably over time or as a summative community practice or behavior dictated by diffusion waves.

Another possible source of bias arises from the fact that some proximate factors that influence infant mortality, such as environmental contamination and nutrient deficiency (according to Mosley and Chen),were not available in the ZDHS [18]. Although presence of various morbid conditions prevalent in this population such as HIV infection, malaria and tuberculosis as well as nutritional deficiency were not concurrently examined as possible co-determinants, it is likely that that these observed population metrics will remain unchanged. In the absence of such data which was not analyzed concurrently in this study as it was not the main focus, we support the argument that the observed determinants can be a proxy of real situation, and selective differential morbidity influences could only to some extent represent a small proportion of the changes observed when population date as this is used. Another limitation may be due to the fact that questions were only asked for the last birth. This means that if a woman had more than one birth within the reference period (the other births) she was not asked these questions and this could have led to insufficient evidence of these factors associated with neonatal mortality.

Notwithstanding the presence of these potential biases, we are still persuaded to believe that if selection and information biases were present, there effect on cascading and identifying a battery of determinants for population-based neonatal mortality was very minimal because the influence of common good dynamics as already explained. In this regard, we believe that the presence of such selection biases due to non-participation, were unlikely to be an important factor explaining the presence of neonatal mortality determinants observed in this population. However we also argue that our findings could have been stronger if patterns and trends of these determinants were examined instead, a fact we acknowledge openly. This we believe would not just improve our estimates but would make external validity considerations more plausible. External validity is a critical challenge when data from selected communities are extrapolated to the whole population. Nonetheless we are persuaded that the determinants of neonatal mortality found in the data examined may approximate well with those of the rest of general population. Firstly, the structure of any Demographic and Health Survey approximate very closely to a census thus providing the basis for many national estimates. Secondly, if hospital based data was used, this tends to over-estimate such demographic parameter like mortality and may thus amplify or show skewedness in the pattern of determinants present. Thirdly, and in this regard, our estimates could be argued to have under-estimated not just the mortality burden but also the effect size of the determinants. If this assertion or argument is held, finding such a high burden of neonatal mortality is disturbing and the observed determinants could be viewed as a first step in informing preventive strategies although it may not tell the whole story for the general population. One factor that was found to be associated with neonatal mortality (and not surprisingly so) was the quality of antenatal care. The results strongly showed that quality antenatal care, which in this case was crudely equated to the number of antenatal care visits, was associated with neonatal mortality in a dose-response fashion. Antenatal care visits made four times and above improved the protective effect of neonatal death. This underscores the fact that in such low income communities basic antenatal care is an important determinant of neonatal mortality as described elsewhere where its effect on maternal mortality rate when practiced as one of the basic components of maternal care on which the lives of mothers and babies depend, has been described [19].

ANC attendance is a factor that can be examined from various angles. A second angle is to treat it as a proxy for quality care, when what is done during ANC or where it is done is examined in light of association with neonatal mortality. Finding that mothers who had their antenatal care at a government health facility had reduced odds of neonatal mortality than those whose mothers had antenatal care at home suggested differential ANC care parameters. A further analysis by place of delivery revealed that delivering at a government health facility and/or a private health facility tended to have increased odds of neonatal deaths compared to delivering at home. This contrasts evidence existing in literature illustrating credence to the vital role that the place of delivery plays in neonatal survival. This is further supported by the 2005 World Health Report which states that giving birth in a health facility (not necessarily a hospital) with professional staff is safer by far compared to doing so at home [1]. In another study conducted in Tanzania another country neighboring and similar to Zambia, delivery outside a health facility remained a significant risk factor for neonatal mortality [20]. Increased neonatal mortality risk was associated with deliveries in government hospitals compared with home deliveries in the rural areas. Similar patterns have been reported in other countries [21, 22]. One explanation for this could be alluded to the selection bias that arose as a result of referral of pregnant women with severe pregnancy complications to these government facilities [23].

Although our results were insignificant and thus could be by chance, they may however indicate the tendency to deliver at such facilities when high risk pregnancies have been identified or indeed when there is a mere perception of risk by someone else through either a community or health service referral system. This may mean selecting potentially high risk cases to attend facility services thus artificially increasing the likelihood of referring these high risk cases which generally have higher mortality likelihood. It also seems this was largely applicable to rural areas. This is because when data was dis-aggregated, delivering in a government health facility was associated with reduced odds of neonatal deaths in urban areas. This further strengthens the hypothesis of self-selecting high risk cases or the mere abundance of high risk cases in rural areas irrespective of access to health facilities. The risk dimensions in rural areas in Zambia may thus be a complex phenomenon dictated by an array of interrelated but differentially effecting forces on child survival. The summary is of this seems to be in rural Zambia neonates are at risk irrespective of place of delivery. The reasons for this need further examination so as to generate information that may be critical to minimize or remove this risk.

Another related individual factor that was examined was birth weight. Low birth weight and over weight in this study were probably the strongest and most consistent determinant found to be associated with neonatal mortality as has been observed in other populations with similar settings [7, 24]. This was the case irrespective of mother's age, place of antenatal care and place of delivery as well as educational attainment of the mother. In Bangladesh it has been reported approximately 75 percent of neonatal deaths are associated with low birth weight attributed to preterm birth rather than small for gestational-age infants [25]. Unfortunately, we were not able to differentiate between preterm and small for gestational-age infants in this study. This finding supports historical evidence that have shown that low birth weight between 2 000 and 2 499 grams are four times more likely to die during first 28 days of life than infants whose weight lies between 3 000 and 3 499 grams [26]. The need for system responses aiming to manage such low birth weight babies is not just challenging for urban areas but pause such a mammoth task in rural communities which forms bulk of the population in low income countries. Generating such community based metrics is one way to enforce such responses despite the limiting resources in these populations. If this is true we further argue for more responsive data generation and monitoring cycles which are community based than the three to seven year practiced DHS cycles. One such approach worth considering is the use of Lot Quality Assurance Sampling (LQAS) which has been attempted recently [27].

Other than health system and individual factors, selected community factors were examined too such as traditional delivery approaches. Assistance during delivery by a relative/friend and having no one to assist were associated with increased odds of neonatal death than being assisted by a skilled health professional. Nevertheless, the presence of a skilled birth attendant, either a doctor/nurse/midwife, is important to ensure appropriate management of the delivery process and to prevent fatal events attributed to delivery-related complications [1].

## Conclusion

In conclusion, we have reported high burden of neonatal mortality and associated determinants in this population. This burden may be a pointer to either failures in past child survival strategies or limitations in existing system responses. The child survival strategies in Zambia are driven by global agenda and are not only complex but they also used multiple approaches that may not be driven by local contexts such as this population based data to inform the interventions. We hypothesise that using clinic based child survival data may have limited responses in the past and lacked more informed parallel survival strategies grounded in a combination of system, individual and community or population based data. The burden observed among predominantly rural suggest a need to refocus and re-package strategies to target selected populations. Unless this is done, existing child survival strategies currently being spearheaded, might remain “irrelevant and inoperable” to these groups whose poor economic and social conditions create an environment that might exacerbate their states. In this regard we further argue that poverty reduction programmes, including strategies to increase maternal educational attainment, are therefore to be seen as necessary components of effective child survival strategies. Furthermore, these findings point to a need for a comprehensive surveillance system to continually capture patterns and trends of these and other potential determinants of child survival.

Lastly but not the least, further research is needed to examine the exact mode of operation through which the stated factors exert their influence on neonatal deaths. Notably, the observed burden was less in urban areas suggesting that that prevention works, and it should continue to be given “highest priority” among all other strategies. However, and although understanding social dynamics associated with this problem was not the focus of this study, we still argue that what works and in what contexts must be known. Examining and understanding such local and indigenous knowledge systems could have a huge potential to inform interventions which in turn could sustain setting driven and socially acceptable programming.
